# Spatial Dispersal of Epstein–Barr Virus in South America Reveals an African American Variant in Brazilian Lymphomas

**DOI:** 10.3390/v14081762

**Published:** 2022-08-12

**Authors:** Paula Alves, Marcella Larrate, Aruanã Garcia-Costa, Paulo Rohan, Bianca Ervatti Gama, Eliana Abdelhay, Edson Delatorre, Rocio Hassan

**Affiliations:** 1 Laboratório de Oncovirologia, Centro de Transplante de Medula Óssea, Instituto Nacional de Câncer “José Alencar Gomes da Silva” (INCA), Ministério da Saúde, Rio de Janeiro 20230-130, Brazil; 2 Laboratório de Células Tronco, Centro de Transplante de Medula Óssea, Instituto Nacional de Câncer “José Alencar Gomes da Silva” (INCA), Ministério da Saúde, Rio de Janeiro 20230-130, Brazil; 3 Departamento de Biologia, Centro de Ciências Exatas, Naturais e da Saúde, Universidade Federal do Espírito Santo, Alegre 29500-000, Brazil

**Keywords:** LMP-1, Epstein–Barr virus, EBV diversity, phylogeography, South America

## Abstract

Epstein–Barr virus (EBV) is a saliva-borne ɣ-herpesvirus associated with benign and malignant lymphoproliferation. EBV-mediated tumorigenic mechanisms are not fully understood and may be related to viral genetic variations. In this work, we characterize the genetic diversity of EBV from Brazil, assessing 82 samples derived from saliva from asymptomatic carriers (*n* = 45), biopsies of benign reactive hyperplasia (*n* = 4), and lymphomas (*n* = 33). Phylogenetic and phylogeographic analysis of the entire coding region of the LMP-1 was performed. Additionally, type 1/type 2 distinction by the EBNA3C gene and Zp variants were evaluated. Our results revealed a high diversity of EBV in Brazil, with the co-circulation of four main clades, described here as: Mediterranean (40.2%, *n* = 33), Raji/Argentine (39%, *n* = 32), B95-8 (6.1%, *n* = 5), and Asian II (1.2%, *n* = 1). The Raji/Argentine and Mediterranean clades were the most prevalent in South America (45% and 28%, respectively). The Raji/Argentine clade was associated with polymorphisms I124V/I152L, del30 bp, and ins15 bp (*p* < 0.0001, to all clades) and with a high haplotype diversity related to EBV type and Zp variants. We found that a Raji/Argentine subclade spread primarily from Brazil and later to other South American countries. Although no LMP1 variant has been directly associated with disease, the Raji/Argentine clade was predominantly clustered with lymphomas (61%) and the Mediterranean clade with non-malignant cases (59%) (*p* = 0.1). These data highlight the high genetic diversity of EBV circulating in Brazil, calling attention to a Raji-related variant with great recombination potential in Brazilian lymphomas.

## 1. Introduction

Epstein–Barr virus (EBV) is a human herpesvirus present asymptomatically in more than 90% of the adult population; in some cases, it is causally associated with lymphoproliferative diseases and malignancies. EBV contributes to up to 1.5% of cancers worldwide, including classic Hodgkin lymphoma (cHL), Burkitt lymphoma (BL), malignant lymphoproliferative disorders in immunocompromised individuals (post-transplant and AIDS-associated), T-cell lymphomas, nasopharyngeal carcinoma (NPC), and gastric carcinoma [1]. The epidemiology of EBV-associated diseases is complex and presents an unusual worldwide geographic distribution, depending on several aspects including age, sex, ethnicity, culture, level of socioeconomic development, and immunogenic factors [2,3,4,5].

EBV is mainly transmitted by saliva and can stimulate and establish latent infection in B lymphocytes that persists during the host’s lifespan [6]. During life, immunocompetent individuals are usually asymptomatic carriers with EBV persisting silenced in peripheric blood at low loads, although transient shedding in saliva may occur with varying viral load [7]. In EBV-associated malignancies, the virus is clonally present in virtually all tumor cells exhibiting different transcription programs, known as latency profiles, which are capable of deregulating cellular signal transduction pathways, favoring cell stimulation and proliferation, consequently ensuring cell survival [1].

EBV possesses a large dsDNA genome, and latent EBNA genes determine the significant component of genetic diversity, thereby classifying EBV into types 1 and 2 [8]. Type 1 is more efficient than type 2 at proliferating and immortalizing B lymphocytes. While type 1 is the prevalent type worldwide, type 2 shows high frequencies in sub-Saharan Africa and in HIV+ individuals. Thus, the types are more associated with specific groups or areas than with a disease *per se*. However, an association of type 1 and V3 variant haplotypes with EBV+ malignancies has been reported [9,10,11,12]. Single nucleotide polymorphisms in the lytic gene BZLF1 (Zp) promoter characterize the V3 variant, and it has recently been suggested that these confer a functional increase in lytic reactivation [10].

Genetic variations in other latent genes have been used to classify EBV diversity, to differentiate geographically restricted viral factors from specific tumorigenic-associated processes [13,14,15,16,17]. Early efforts led to positive findings related to deletions within the C-terminal domain of LMP-1 oncogene associated with European cHLs and Asian NPCs. In line with this finding, the CAO LMP1 variant, isolated from a Southeast Asian individual diagnosed with NPC and carrying these deletions, showed to be more oncogenic in vitro than the prototype B95-8 strain, and was able to develop a tumor in vivo [13]. Subsequently, Guiretti et al. suggested a pathogenic role for the LMP-1 variant harboring 30 bp deletion (del30) in cHL cases from South America [14].

In an overexpression model, LMP-1 demonstrated the ability to transform rodent fibroblasts in vitro and to cause tumors in vivo. In this way, LMP-1 was the first EBV oncoprotein related to EBV-mediated transformation [18]. LMP-1 can be highly expressed in various EBV-associated tumors, but not usually in EBV-infected non-neoplastic tissues [19]. Thus, LMP-1 has been observed to modulate essential pathways for cell signaling and survival, such as resistance to apoptosis, angiogenesis, and immunomodulation [20]. LMP-1 mimics the cellular transmembrane protein CD40, acting like a constitutively active receptor independently of ligand binding [21].

LMP-1 consists of three domains totalizing 386 amino acids (aa) and 63kD, including the short cytoplasmatic amino-terminus (N-ter, 1–24 aa), six transmembrane domains (TM, 25–186 aa), and a carboxy-terminal signaling domain (C-ter, 187–386 aa). Each domain possesses different characteristics and functions during the viral replicative cycle and cellular transformation, displaying a high intra-host variability [22,23]. Some LMP-1 polymorphisms, when compared to the B95-8 strain [24], have relevant epidemiological importance. The *Xho*I restriction site in the N-ter domain has been associated with geographically restricted strains or specific ethnic groups [25]. The I124V/I152L and F144I/D150A/L151I polymorphisms at the TM domain appeared to increase NF-kB activity in vitro. Despite its pathogenic potential, these polymorphisms were described in an HIV+ Swiss population and in children from Argentina with benign and malignant conditions [26,27]. Of note, no association with malignancies was found. Furthermore, the LMP-1 C-ter domain shows high genomic instability, and is characterized by several polymorphisms and mutation hotspots, including the insertion of 15 bp (ins15) that encodes a Janus Kinase 3 (JAK3) motif [28,29] and the deletion of 30 base pairs (del30). The del30 has been associated with various malignant cases worldwide [13,14], and was recently suggested as favoring immune escape in the context of LMP-1 variants in cytokine production in HL’s cells in vitro [30].

Analysis of the genetic diversity present in LMP-1 allowed the classification of EBV in several strains or types [29,31]. However, the association between pathological conditions and these types is still controversial [32]. Gantuz et al. [27], evaluating the LMP-1 genetic diversity in South America through phylogenetic analysis, reported a new LMP1 variant. This variant was named Argentine due to a high prevalence among the assessed Argentinian population, regardless of the presence of malignancies. The LMP1 Argentine clade was closely related to the Raji clade, which had the same common ancestor, although it harbored I124V/I152L and del30 polymorphisms, absent in the Raji strain. In Brazil, despite the association of del30 and ins15 with Brazilian lymphomas [14], a recent study evaluated the full length of the coding sequence of the LMP1 gene, and its promoter demonstrated a pattern of polymorphisms associated with Brazilian BL; a further close relationship between Brazilian and African strains was suggested [9,17].

Since the worldwide distribution of various EBV strains likely reflects human migration over the past few centuries [28,33], it is expected that historical human migration events may explain viral gene pools isolated in other populations [27,33]. However, in the worldwide EBV genetic diversity landscape, South America is still poorly understood, lacking representation of various countries from this region [16,27,34]. Moreover, Brazil has a highly admixed population with diverse socioeconomic status, and beyond that, it has been reported to demonstrate different epidemiological patterns related to EBV-associated diseases; nevertheless, incipient data suggests the genetic variability of EBV [9,14,17].

This work intends to describe the molecular diversity of EBV in Brazil and its regional context in South America, through genetic characterization of LMP-1 variants from natural carriers, EBV-associated hematological pathologies, and the relationship of EBV with other viral genotypes. In addition, we reconstructed the dynamic of viral migrations of the LMP-1 main clade circulating in South America, to contribute to the growing knowledge of the genomic evolution of EBV.

## 2. Materials and Methods

### 2.1. Study Population

A total of 45 EBV+ saliva samples from asymptomatic carriers (AC) residing in Brazil were collected between March 2018 and April 2019 from the microregion of the Rio de Janeiro state. Saliva was obtained by participants dribbling 5 mL into a 50-mL plastic centrifuge tube. After agreeing to participate in the study, demographic data of the ACs were obtained at sample collection. The information collected included age, sex, country of birth and residence up to 18 years old (yo), current country of residence, and cancer diagnosis. Although the serum status of EBV infection was not evaluated, EBV seroconversion in Brazil, as in Latin America, commonly occurs in early childhood [34,35,36]. Thus, instead of place of residence, we considered the place of origin up to 18 yo as the geographical representation of the EBV origin (Supplementary Material Table S7).

We used fresh-frozen biopsies of lymph node EBV+ of benign reactive lymphoid hyperplasia (RLH, *n* = 4), and malignant cases of classic Hodgkin lymphoma (cHL, *n* = 26) and Burkitt lymphoma (BL, *n* = 7) from the pathological cases. These patients were diagnosed between 1995 and 2007, within the Integrated Division of Pathology (DIPAT) at the Brazilian National Institute of Cancer (INCA), located in the capital of Rio de Janeiro state. The diagnoses followed the morphological and immunohistochemical criteria established by WHO [37]. The demographic data of the patients, obtained from medical records, included age and sex.

The study was carried out following the approved guidelines, and measures were taken to provide confidentiality for patients and asymptomatic donors, and to assure security of the data obtained. The Research Ethics Committee of INCA approved this study (CAAE approval 53571116.4.0000.5274).

### 2.2. DNA Extraction, Amplification, and Sequencing

Genomic DNA was extracted from fresh lymph node biopsies using a QIAamp DNA Mini kit (QIAGEN, Hilden, Germany), the approach followed the manufacturer’s protocol. The extraction of whole DNA from the saliva collected in sterile tubes was carried out by an in-house salting out method, aiming to achieve fast and low-cost processing for a large number of samples. The in-house method consisted of: (i) centrifugation of 500 µL of saliva with 17,000× *g* for 5 min, (ii) the supernatant was discarded and (iii) 500 µL of 50 mM NaOH was added, (iv) then the mixture was homogenized with aid of a vortex mixer, and incubated for 5 min at 95 °C. (v) Subsequently, the sample was neutralized with Tris-HCl (1 M, pH 6.8), with volume of 10% *v*/*v* NaOH. (vi) Then, the mixture was centrifugated by 8609× *g* for 5 min. Then, (vii) the supernatant was transferred to a new tube and the precipitate was discarded. The supernatant containing DNA was stored at −20 °C. The quantification and purity of all extracted DNA samples were measured using Nanodrop^TM^ 8000 (Thermo Fisher Scientific, Waltham, MA, USA). The products were evaluated in 2% agarose gel containing ethidium bromide 0.5 µg/mL.

EBV type 1 or 2 was determined by EBNA3C gene amplification as previously described [38]. The complete sequence of the LMP1 oncogene of 1390 bp was amplified by nested PCR separately targeting the three domains of the protein (N-ter, TM, and C-ter) as previously described [29]. Amplification of 333 bp of the promoter region (−221 to +12) of Zp was performed as previously described [39]. To allow DNA sequencing reaction by the Sanger method, the PCR products of LMP1 and Zp regions were purified using QIAquick PCR purification (QIAGEN, Hilden, Germany) following manufacturer’s instructions. Then, the products were sequenced using Big Dye Terminator v.3.1 (Applied Biosystems, Waltham, MA, USA) in an automated Genetic Analyzer ABI 3130xl (Applied Biosystems, Waltham, MA, USA) using the same PCR primers.

### 2.3. Detection of Polymorphisms, Phylogenetics and Phylogeography Analyses

The new LMP1 sequences generated were assembled using sequence B95-8 (GenBank number NC_007605) as reference. The sequence quality was evaluated and the contigs of the N-ter, TM, and C-ter domains were assembled using SeqMan Pro (DNASTAR^©^, version 11.1.0, Madison, WI, USA). The polymorphisms that characterize the V3 variant of Zp were evaluated using the prototype type 2 AG876 strain (NC_009334) as reference. The polymorphic positions to define the V3 variant in Zp were −141 (A > G), −106 (A > G), −100 (T > G) when related to the B95-8 strain [39].

For initial classification and identification of the main EBV clades present in our sample, we added the newly generated LMP1 sequences to a dataset containing LMP-1 reference sequences from EBV+ cell lines and geographic prototypes, recognized for characterizing different variants [29], and available in Genbank (Supplementary Material Table S8). The alignment was built using the MAFTT algorithm [40] and examined by MEGA 7 [41] to identify introns, indels, and repetitive regions. The alignments were employed to generate phylogenetic trees using the Maximum likelihood (ML) algorithm performed by PhyML [42], and the branch support was evaluated by the approximate likelihood ratio test (aLRT). The phylogenetic trees were assessed and annotated in the FigTree software (http://tree.bio.ed.uk/software/figtree/ (accessed on 23 February 2022). The GTR+G nucleotide substitution model was selected through the JModelTest 2 program [43].

For the global phylogenetic analysis, sequences of the entire gene of the LMP-1 oncoprotein over 1100 bp of extension, available in Genbank until August 2022, were added in our previous sequence dataset, with geographical and time of sampling information (Supplementary Material Table S9). The BEAST v1.10 [44] coupled with the BEAGLE library [45] was used to reconstruct the phylogeographic history of the Raji/Argentine clade identified in this study. Discrete phylogeographic analysis was performed using reversible (symmetric) or nonreversible (asymmetric) discrete phylogeographic models [46]. The evolutionary process was estimated using the best-fit nucleotide substitution model (TN93 + G), a relaxed uncorrelated lognormal, or a strict molecular clock model [46]. The best molecular clock or phylogeographic model was chosen using the log marginal likelihood estimation (MLE) based on path sampling (PS) and stepping-stone sampling (SS) methods [47]. The MCMC run had 5 × 10^7^ generations and convergence (effective sample size > 200) was inspected using TRACER (version 1.7, Rambaut et al, Edinburgh, UK) [48] after discarding 10% burn-in. The substitution model was selected through the JModelTest 2 program [43].

### 2.4. Statistical Analysis

The associations between categorical variables were tested using Pearson’s chi-square test or Fisher’s exact test. For continuous variables with non-parametric distribution, the median value and the minimum and maximum values were used. A *p*-value < 0.05 was indicative of statistical significance in the tests. All statistical analyzes were performed using the SPSS software, version 20.0 (IBM, Armonk, NY, USA).

### 2.5. Data Accession Numbers

All LMP1 sequences obtained in this study were submitted to the GenBank database. The assigned accession numbers were ON596440–ON596521 (Supplementary Material Table S7).

## 3. Results

### 3.1. Characteristics of the EBV+ Population

The EBV+ sample analyzed in this study comprised a total of 82 individuals residing in Brazil, of whom 49 (59.8%) were asymptomatic carriers and benign cases (ACB), and 33 (40.2%) were malignant cases (MCs). The ACB group consisted of individuals with a median age of 26 years old (yo) and sex ratio M:F = 1:2. Regarding MCs, individuals carrying lymphoma cases presented a median age of 26 yo and sex ratio M:F = 2.5:1. As stated above, we considered the location as the place of birth and residence up to the age of 18 years, assuming that the first EBV infection occurred in childhood. Thus, two asymptomatic samples collected in Brazil were considered to originate from Africa and one was of Peruvian origin.

EBV genotyping by the EBNA3C gene in the ACB group characterized 79.6% belonging to EBV type 1 and 20.4% to type 2. Regarding the MC group, 90.9% of the sequences belonged to type 1 and 9.1% to type 2. The prevalence of the two EBV types observed between the two groups was not statistically different (*p* = 0.22).

### 3.2. LMP1 Phylogenetic Analysis

**Figure 1:**
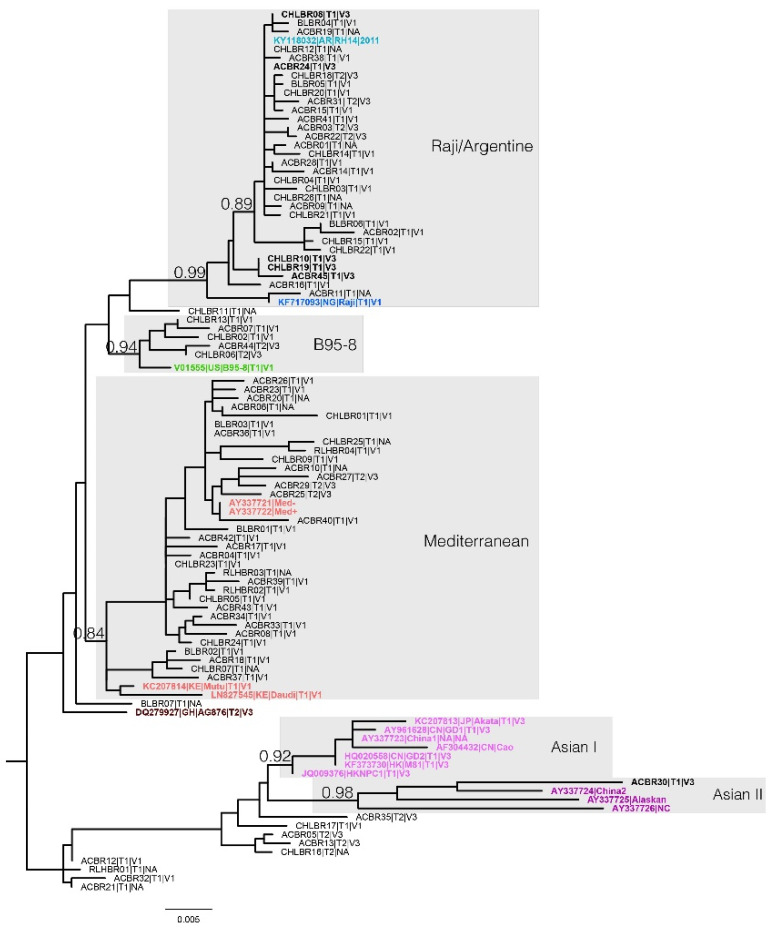
Maximum likelihood phylogenetic tree of the complete coding region of LMP1 oncogene sequences from asymptomatic carriers and pathological cases. Reference sequences included in the analysis belong to variants: B95-8 (green); Med+/−, Mutu, and Daudi (red); AG876 (dark red); Akata, GD1, China1, Cao, GD2, M81, and HKNPC (pink); China2, Alaskan, and NC (purple); Raji (blue); and Argentine (light blue). Five highly supported main clades harboring reference sequences (grey shadows) were identified. The tip labels of our samples represent: case type (AC: asymptomatic carrier; cHL: classic Hodgkin lymphoma; BL: Burkitt lymphoma); EBV type (T1 for type 1 or T2 for type 2); and Zp variant (V1 or V3). The samples carrying haplotype T1 + V3 are named in bold. aLRT support values are indicated at the main branches, and the branch lengths are to scale with the lower bar indicating nucleotide substitutions per site. The tree was rooted at the midpoint.

The ML phylogenetic classification of the LMP1 entire coding region indicated with high support (aLRT ≥ 0.84; Figure 1 and Supplementary Material Table S1) the presence of four clades circulating in Brazil. Approximately 79.2% of Brazilian EBV sequences clustered into two main clades: 40.2% into one clade which gathered the Med+/−, Daudi, and Mutu reference strains (aLRT = 0.84) named here as Mediterranean, while 39% clustered with the Raji strain and Argentine variant (aLRT = 0.99). Furthermore, 6.1% of sequences clustered with the B95-8 reference strain (aLRT = 0.94), and 1.2% of sequences to the Asian II clade, which harbored China2, Alaskan, and NC reference strains (aLRT = 0.98). The remaining sequences (13.4%) did not group into well-defined clades with other reference strains; therefore, this method could not classify them (Figure 1). Despite the high diversity in the Brazilian samples, there was no association between malignant cases and the four clades defined in this work (*p* = 0.3, Supplementary Material Table S2). On the other hand, when we compared only the two main clades circulating in Brazil, which accounted for 79% of the sampling, interestingly, we observed that the clade Raji/Argentine mostly clustered sequences derived from MCs (61%), while the Mediterranean clade (59%) mainly grouped samples from the ACB group (*p*= 0.1, Supplementary Material Table S2).

### 3.3. Association of the Brazilian LMP1 Variants with Polymorphisms

**Figure 2:**
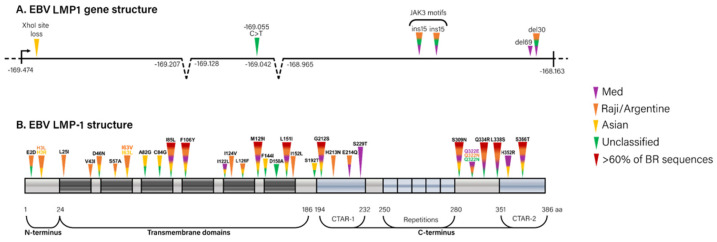
Genetic map of the LMP-1 polymorphisms found in Brazil related to the prototype B95-8 strain (GenBank number NC_007605). (**A**) Structure of the *LMP1* gene highlighting the position of *Xho*I site loss, SNPs, insertions, and deletions. The dotted region denotes the intron positions. The color of the triangles represents the clade associated with the polymorphism, following the legend. (**B**) Structure of the LMP1 protein showing the domains. The triangles indicate the approximated position of each analyzed polymorphism, colored according to the associated clade (legend on the right). The color of the polymorphism is associated with the most prevalent clade (black indicates no association).

To understand the diversity identified in the phylogenetic analysis, we evaluated the relationship of the LMP-1 polymorphisms (Figure 2) with the main clades (Supplementary Materials Figures S1 and S2, Tables S3 and S4). The loss of the restriction site of *Xho*I (N-ter) and the change C > T in position 169,055 (TM domain) were limited to specific groups of sequences, the first to the Asian II clade harboring only one sequence, and the second only to the sequences without classification (*p* < 0.0001, for *Xho*I and C > T, separately). The polymorphisms I124V/I152L, also in the TM domain, were present exclusively in Raji/Argentine clade (*p* < 0.0001), with high frequency (93.8% of the sequences). The polymorphic triad F144I/D150A/L151I was identified only in sequences that did not fall within the five main clades. The change L151I alone was detected mainly in the Raji/Argentine clade (100% of the sequences), to a minor extent in the Mediterranean (48.5%) clade, and in two sequences without classification (18.2%), while the B95-8 and Asian clades did not display this change (*p* < 0.0001). There was no statistical difference in the number of repeat units of rep33 in different clades, with a median number of repeats of four or five (*p* = 0.2). The frequency of ins15 was higher in the Raji/Argentine (87.5%) and B95-8 (100%) clades when compared with Mediterranean (12.1%) and Asian (absent) clades (*p* < 0.0001). In this way, the ins15 was the only polymorphism associated with malignant clinical outcomes (Supplementary Material Table S4) since it was present in 66.7% of MC cases (*p* = 0.007). Del30 was identified with higher frequencies in the Raji/Argentine (71.9%) clade and, curiously, in the majority (90.9%) of sequences without classification (*p* < 0.0001). Despite the surprising identification of del69, its presence was found exclusively in the Mediterranean clade (9.1%), and it was not a statistically significant marker of this clade (*p* = 0.64). When we focused our analysis on the two main LMP1 clades found in Brazil (the Mediterranean and Raji/Argentine), 61.5% of the MCs were clustered in the Raji/Argentine clade, harboring the polymorphisms I124V/I152L (100%), L151I (100%), ins15 (81.3%) and del30 (69%). In contrast, the Mediterranean clade clustered mostly ABC samples (59%) with a total absence of I124V/I152L and ins15 mutation, and lower frequencies of the polymorphisms L151I (43.5%) and del30 (4%) (*p* < 0.01 to each, Supplementary Materials Tables S3 and S4).

### 3.4. Relationship of Brazilian LMP1 Variants with EBV Type and Zp Variants

We also assessed EBV type by EBNA3C gene and Zp variants, in a preliminary attempt to understand the haplotypic relationship of LMP1 variants classified in this present work. Surprisingly, the Brazilian samples evaluated (*n* = 63) showed a high haplotypic diversity (Figure 1 and Supplementary Material Table S3). The Mediterranean clade gathered mostly the haplotype Type1 + V1 (88.9%), and clustered sequences harboring Type2 + V3 (11.1%) to a lesser extent. Strikingly, the Raji/Argentine clade was associated with the highest haplotypic diversity, being the only clade that harbored the three types of haplotypes already described related to EBV type and Zp variant: Type1 + V1 (65.4%), Type1 + V3 (19.2%), and Type2 + V3 (15.4%). Contrasting to the Mediterranean clade, the haplotypes that belonged to Raji/Argentine clade did not form specific clusters. A small fraction of analyzed sequences belonging to the B95-8 clade harbored the Type1 + V1 (60%) and Type2 + V3 (40%) haplotypes. Finally, the only sequence from our sample that branched into the Asia II clade revealed the Type1 + V3 haplotype. On top of all these observations, it is remarkable that 83.3% (five of six) of the LMP1 strains with the Type1 + V3 haplotype belonged to the LMP1 Raji/Argentine clade (*p* = 0.051 when compared with Mediterranean and B95-8 clades), regardless of disease association (Supplementary Material Table S4).

### 3.5. EBV Diversity and Spatial Dispersal in South America

**Figure 3:**
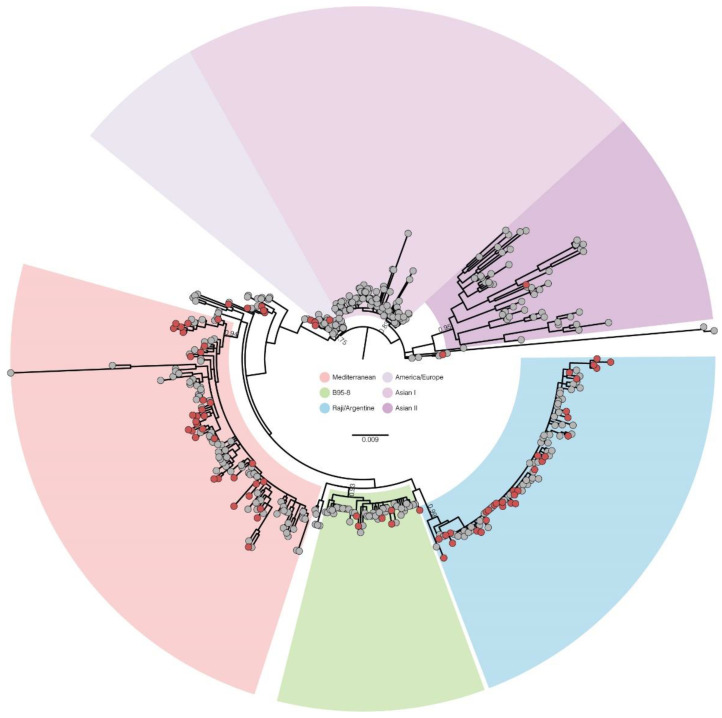
Maximum likelihood phylogenetic tree of the LMP1 gene from the Brazilian samples in this work (*n* = 82, red circles) and those available in the GenBank database (*n* = 472, gray circles). Six main clades were observed, marked by different shadings, according to the legend at center (Mediterranean—grouping the references Med+, Med− Daudi, and Mutu; Asian II—grouping the references HKNPC1, M81, Cao, Akata, NPC, and China1; Asian II—grouping the NC, Alaskan, and China2 references; America/Europa—grouping sequences from different countries without a reference strain. The aLRT branch support values are displayed in key branches. Branch lengths are scaled with the bar indicating nucleotide substitutions per site.

To put the newly identified EBV LMP1 sequences into a global evolutionary context, we conducted a ML phylogenetic analysis of the new 82 LMP1 sequences generated in this work, and 472 complete LMP1 sequences available on GenBank. The sequences were distributed into six main clades with high support (≥0.75 aLRT branch support, Figure 3 and Supplementary Material Table S5). These clades were named as follows: Mediterranean, clustering 24% of the sequences, with aLRT support = 0.94—including the references Med+, Med-, Daudi and Mutu; Raji/Argentine, grouping 19% of the sequences, with aLRT = 0.99—grouping the Raji and Argentine references; B95-8, with 9% of the sequences and aLRT support = 0.93—including only the B95-8 reference strain; Asian-I, with 22% of the sequences, with aLRT = 0.83—clustering the references HKNPC1, M81, Cao, Akata, GD2, and China1; Asian-II, with 10% of the sequences, aLRT = 0.95—grouping NC, Alaskan, and China2 references; and an America/Europe clade with 6% of the sequences, with aLRT support = 0.75—with no reference strains. A total of 73% of the LMP1 sequences from South America clustered within the Raji/Argentine (45%) or Mediterranean (28%) clades, characterizing these as the two main lineages circulating in the continent. Other sequences from South America clustered in minor clades related to the B95-8 strain (7%) or within the America/Europe clade (2%). Regarding Asian clades, 7% of South America sequences branched inside the Asian-II clade. Some sequences from South America (10%) representing Brazil and Argentina did not cluster in any of the six main clades mentioned and could represent recombinant lineages or underrepresented lineages. Interestingly, a dyad of Brazilian and Argentine sequences clustered with high support (aLRT = 0.99), suggesting an exclusive lineage from South America, albeit at low prevalence.

**Figure 4:**
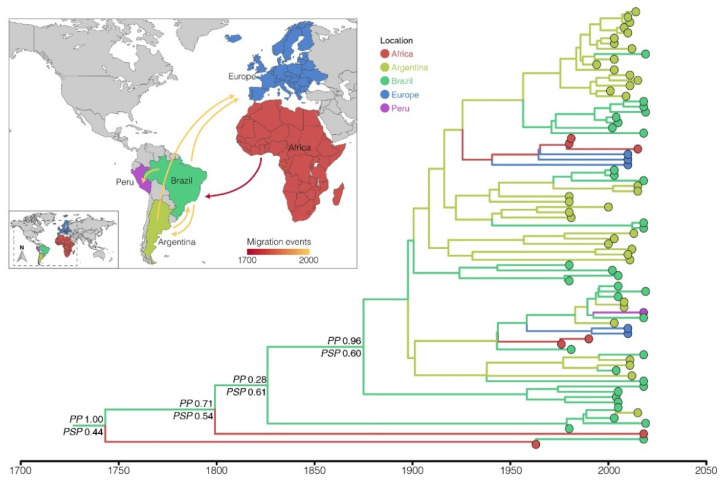
Dispersal of the Raji/Argentine subclade circulating in South America. Bayesian MCC time-scaled discrete phylogeographic tree for the Raji/Argentine clade. The colors of the branches represent the most likely location of its descending nodes, with the key colors shown on the legend. The posterior probabilities (PP) and the posterior state probabilities (PSP) are annotated in the basal nodes. All horizontal branch lengths are drawn on a scale of years. The inset represents the viral migrations routes of the Raji/Argentine clade based on the location probability of the MCC tree. The arrows represent the viral migrations routes of Raji/Argentine clade based on the location probability of the MCC tree, and are colored according to time as indicated by the legend.

Aiming to understand the geographic origin and spatial spread of the most prevalent clade in South America, Bayesian phylogeographic analysis was performed to reconstruct the dynamics of viral migrations of the Raji/Argentine clade (Figure 4). The Bayesian phylogeographic reconstruction using the strict molecular clock and the nonreversible (asymmetric) discrete phylogeographic models (Supplementary Material Table S6) estimated the LMP1 gene median molecular clock rate at 3.8 × 10^−5^ [highest probability density (HPD): 1.9 × 10^−5^–5.9 × 10^−5^] substitutions/site/year. According to the reconstructed phylogeny, the EBV Raji/Argentine strains circulating in South America (Brazil, Argentina, and Peru) have Brazil as their primary source of spread. The phylogeographical analysis pointed to Brazil as the place of origin of the Raji/Argentine clade (posterior location probability = 0.44). However, this hypothesis is probably biased by the high number of Brazilian sequences in our sample (~40%). The alternative hypothesis points to Africa as the origin of this clade (posterior location probability = 0.25), despite the deficient proportion of African sequences in our Raji/Argentine subset (~8%). In this more epidemiologically realistic scenario, the Raji/Argentine clade circulating in South America originated through a viral introduction from Africa to Brazil between the 17th and 19th centuries. Subsequently, Brazil (and to a lesser extent Argentina) acted as the source of this strain to other countries on the American continent (Argentina and Peru) and in Europe (Figure 4). Although our phylogeographic reconstruction indicates the return of this strain from Brazil to Africa, these migration events may represent artifacts from the low sampling of the viral genetic diversity of the African continent.

## 4. Discussion

Several studies have demonstrated that there may be a link between genetic variation in EBV and associated diseases [49]. In this way, viral genetic polymorphisms could impact the virus–cell interaction and eventually favor development of malignancies [50]. In this context, the LMP1 oncogene has been extensively studied due to its wide intrahost variation and impact on EBV-mediated oncogenesis. Consequently, several LMP-1 polymorphisms with high pathogenic potential in neoplastic cases have been described [9,13,14,26,29]. However, there is no consensus on classifying EBV LMP-1 diversity, and several classification systems have been proposed [9,27,29]. Some of the systems of classifications use a partial or whole LMP1 gene sequence, with or without the promoter region, and often consider a restricted geographical sampling scheme, making it difficult to compare methods [9]. In this work, we adopted a maximum-likelihood phylogenetic approach considering the whole LMP1 gene, coupled with the investigation of essential polymorphisms described in the literature [14,15,25,26,27,28,29] for the genetic classification of our sampling.

To the best of our knowledge, this is the first report of the genetic diversity of the whole LMP1 oncogene from asymptomatic Brazilian carriers and benign and malignant hematological cases. The genetic diversity of the Brazilian LMP1, indicated by our phylogenetic analysis, comprised five highly supported monophyletic clades, two of which represent the most prevalent EBV lineages circulating in Brazil: Mediterranean (grouping Edward’s pattern Med+ and Med-, and the lineages AG876 and Daudi) and Raji/Argentine (grouping the lineage Raji and the Argentine variant). We found that several polymorphisms present in the TM and C-ter domains of LMP1 were associated with the Raji/Argentine clade; including I124V/I152L, able to enhance NF-κb activity in vitro [26]; ins15, which adds an extra JAK3 motif [28]; and del30, found in CAO strain and to another set of Brazilian MC samples [13,14]. Surprisingly, the del69 was detected in Brazil exclusively in asymptomatic carriers. Despite the del69 being primarily associated with NPCs and cHL in other geographic regions [13,51], Brazil lacks data to establish a link with different clinical manifestations, the pathogenic role of this polymorphism has not yet been suggested. Sueur et al. [30] demonstrated different functional roles of del30 and del69, indicating that an LMP1 strain harboring del30 had a lower capacity to stimulate pro-inflammatory cytokine production in comparison with the del69 variant, thus suggesting an immune escape role for the LMP1 del30 variant. Our results reinforce in part the findings of Guiretti et al. [14], which demonstrated that the del30 and ins15 polymorphisms were associated with lymphomas found in patients from Brazil and Argentina.

Although no specific LMP1 clade was significantly associated with patients’ disease status, most sequences clustered within the Raji/Argentine clade corresponded to the MC group, while the Mediterranean clade harbored mainly ACB. Even though we do not have detailed information indicating the ancestry background of the individuals sampled in this present work, this association may have a related sampling bias. Our sampling of tumor biopsies was derived from a Public Health System, and it is known that the majority of patients treated by the public system are of Afro-descendant origin, with a poorer socio-economic situation and a lower level of education [52]. On the other hand, our saliva samples were derived mainly from students and professionals related to the postgraduate academy, and their families. Bearing these considerations in mind, we must not rule out an ethnic-social-economic bias in our sample, which would be in consonance with the current expectations of EBV being related to human migration, and perhaps more associated with specific ethnic populations than disease *per se* [28,33].

The analysis of one LMP1 domain alone may not be sufficient to reflect the total gene diversity or even define its classification into different lineages [9,27]. It is possible that the low description of these variants close to the Raji clade in South America [14,15] may be due to the use of the C-ter region, as the main polymorphisms present in this sequences (I124V/I152L) are located in the TM domain [26]. Previous work by our group, with a different set of lymphoma cases using only polymorphisms of the C-ter domain, did not detect the presence of this variant [14]. It is important to note that the original Raji strain cannot undergo viral production, possibly due to a series of deletions in its genome [53]. Nonetheless, the Brazilian sequences that branched within the Raji/Argentine clade share a common ancestor with the Raji strain and do not seem to be directly derived from it. Even the sequences from our samples that were monophyletic to the Raji strain did not appear to have the same deletions as Raji [54], since these were typed for EBV through the EBNA3C gene, which is found with a large deletion through its almost total genic extension in the original Raji strain. In this way, the abortive lytic phase and lack of production of new particles from the Raji strain may be related to genomic instability in the tumor of origin, or even to the process of in vitro immortalization, which could generate genomic alterations and later affect its functionality. Furthermore, these variants related to the Raji clade were found in great frequency not only circulating in Brazil but also in Argentina [9,27], which characterizes the role of a variant successfully replicating and circulating among different populations.

Lei et al. [9] evaluated for the first time the full length of the LMP1 gene from EBV-infected patients from Brazil, demonstrating that most of the Brazilian sequences clustered closer to the Raji strain, suggesting a close relationship between Brazilian and African EBV strains. Using a larger sampling scheme, Liao et al. [17] confirmed the patterns of polymorphism identified by Lei et al. shared between Brazilian and African BL cases. These polymorphisms included the substitutions I124V/I152L present in all Brazilian BL clustered close to the Raji clade analyzed by Lei et al., as well as all MC samples grouped in the Raji/Argentine clade described in this work. Although these studies have pioneered the description of the EBV diversity present in Brazilian BL cases, both lack representativeness of the EBV present in the Brazilian asymptomatic (or “healthy”) population.

Exploring a set of EBV-infected Argentinian pediatric samples, Gantuz et al. [27] reported a high prevalence of a clade closely related to the Raji strain, with no clear association with the clinical outcome. This clade was characterized by polymorphisms in different domains of the protein, which were absent in the original Raji strain, including I124V/I152L (TM) and del30 (C-Ter), and consequently was defined as the Argentine variant. It is worth noting that these polymorphisms were present in all new sequences from South America described in this work that branched into the paraphyletic clade closest to the Raji strain, which had the Argentine variant as a reference. To understand the spread of the Raji/Argentine clade, which seems to be the most prevalent clade in South America, we conducted a Bayesian phylogeographic analysis. The Raji/Argentine subclade, previously described as the Argentine variant, seems to have originated in Brazil from EBV strains introduced from Africa that are closely related to the Raji reference strain in the LMP1 gene. Therefore, Brazil acted as a staging post in the dissemination of this lineage between Africa and other South American countries, which is reflected in the high diversity of the EBV Raji/Argentine clade in the country.

These results contradict Gantuz et al.’s hypothesis that Argentina is the putative origin of this variant that possibly came from Africa. Historically, Brazil received enslaved African people from almost all the west African coast, numbering three million more than all other countries from Spanish America [55], reinforcing Brazil as being the probable gateway of these African variants in South America. However, more sequences are needed derived from countries on the west African coast, such as Nigeria, Ghana, and Angola, to confirm whether the origin of these variants related to the Raji strain is in Africa, or even in some South American country such as Brazil, which was the primary receptor of Africans in the diaspora. At present, this variant related to the Raji strain is highly present in South Americans, being the main EBV variant circulating in the continent. Our phylogeny demonstrates a high diversity within this Raji/Argentine clade, with the phylogeographic analysis showing that great diversity from a Brazilian base could spread these variants to other South American countries, such as Argentina and Peru, and more recently to Europe. The local evolution of this Raji-related variant in Brazil is evidenced by several sequences that do not carry the Argentine subclade-defining mutations, indicating Brazil as a place where the co-circulation of ancestral and Raji-related lineages may occur.

Our results demonstrate the remarkable genetic diversity of EBV circulating in Brazil, pointing to the importance of the description of its natural diversity in samples from asymptomatic and symptomatic individuals, and the surveillance of mutations that could increase the pathogenic potential of different strains. Along with potential pathogenic LMP-1 polymorphisms, we evaluated the main EBV genotyping by the EBNA3C gene that classifies EBV types 1 and 2. Thus, type-1 EBV was detected in 84.1% of our sampling and type-2 in 15.9%, indicating that the prevalence of EBV types has not changed in the last 20–30 years in Brazil, regardless of clinical outcome [56,57]. The prevalence of EBV types in Brazil is similar to other countries, however, a high diversity of EBV type 2 sequences was identified concerning LMP-1 oncoprotein variants. Notably, the two main clades in our sample, Raji/South American and Mediterranean, encompassed 30.8% and 23.1% of the EBV type 2 sequences. The African strain Raji has a type 1 origin, and the Mediterranean pattern has not been commonly identified as EBV type 2 [16]. These data demonstrate the importance of evaluating geographically diverse sample sets.

In investigating different LMP1 variants in haplotypic association with EBV type and Zp variant, it is essential to highlight that the Raji/Argentine clade showed a high haplotypic diversity when compared with the Mediterranean clade. The presence of Type1 + V3 haplotype was not associated with disease, but it was highly present within the Raji/South American clade of LMP1. Therefore, high haplotypic diversity suggests that the Raji/Argentine clade may harbor variants with high recombination capacity. Although recombination is one of the leading forces that impact the diversity of EBV [58], its evaluation was one of the limitations of our study focused on characterizing the molecular epidemiology of EBV circulating in Brazil. Thus, further work is needed to characterize variants related to the Raji/Argentine clade, which, in addition to being the main variant circulating in South American samples, has polymorphisms in LMP-1 that represent functional gains, as well as being associated with a haplotype with high replicative capacity. The possible effect of the recombination process in the Raji clade in South America has already been proposed by Gantuz et al., since Argentinian sequences related to the Raji clade could have recombined with sequences related to the Asian China1 variant [27]. Here, we suggest that it is possible that a recombination between EBV occurred originating from Native Americans that could inherit polymorphisms related to Asian sequences, and thus related to ancestral human migration from Asia to America thousands of years ago. This hypothesis is strengthened in this present work when we observe sequences, both Brazilian and from other South American countries, without a clear classification in the phylogeny (“unclassified” in our report), were found to be close to clades or sequences of Asian origin. In addition, we were able to identify through the investigation of polymorphisms a relationship of the unclassified sequences with the Asian ones, but probably due to the lack of representation of South American sequences related to Amerindian populations we were not able to increase the strength of this relationship. Therefore, future works using sequences from specific populations of Native Americans, as well as from other countries that helped to shape the highly admixed population in South America, are needed to better understand the origin, dispersion, or even selection of new EBV strains.

Our observed associations between polymorphisms in LMP1 and other genomic regions, like EBNA3C and Zp variants, reinforce the findings from Palser et al. [16] and Zanella et al. [59], suggesting that one gene may not be sufficient to correctly interpret EBV diversity, implying the necessity to assess different viral haplotypes for consistent EBV classification. However, in regions where complete genome studies are not feasible, the separate characterization of some genome targets could represent important biomarkers to study EBV geographic diversity at relatively low costs [60]. Phylogenetic analysis carried out with LMP1 from South America [59,61] highlights the importance of investigating EBV diversity in a geographic region marked by high diaspora and immigration flow, resulting in a mixed ancestry that could impact greater diversity of this ubiquitous virus.

This is an initial work exploring the diversity of EBV in Brazil, and it has several limitations. Most of the samples analyzed were from the Brazilian southeast macro-region; Brazil covers an extensive territory, with different regions home to a highly admixed population with a diverse status of socioeconomic development, which could reflect in different epidemiological patterns of EBV-related diseases [3,4,62]. Further studies are needed to define specificities related to populations from different regions, ethnicities, and socioeconomic statuses to better understand these relationships. Thus, further work with a broader sampling strategy is needed to better understand the molecular epidemiology and spread of EBV strains in our geographic area, including how the intrinsic genetic and ethnographic characteristics of the Brazilian population allowed the adaptation and persistence of the South America variant, which might be associated with neoplasm development.

## 5. Conclusions

Our results highlight the high genetic diversity of EBV epidemics in Brazil, with the co-circulation of at least five phylogenetic distinct LMP1 clades. The Mediterranean and Raji/South America are the main lineages circulating in Brazil, and might be related to ancestry or even distinct clinical outcomes. Furthermore, the Raji/Argentine variant is the central lineage circulating in South America, and we found that Brazil acted as the source for spreading this variant to other countries in South America. The phylogenetic analysis of the whole LMP1 gene could be a promising approach to understanding EBV genetic variants and their potential association with EBV-related diseases.

## Data Availability

The newly generated LMP1 sequences were submitted to GenBank: ON596440-ON596521.

## Supplementary Materials

The following supporting information can be downloaded at: https://www.mdpi.com/article/10.3390/v14081762/s1, Table S1: Phylogenetic classification of population investigated; Table S2: Relationship of LMP1 clades and clinical outcomes; Table S3: Characterization of EBV polymorphisms in the population investigated; Table S4: Relationship of polymorphisms with clades and clinical outcomes investigated; Table S5: Global LMP1 phylogenetic classification; Table S6: Best fit molecular clock and discrete phylogeographic model for the EBV LMP1 Raji/Argentine clade dataset; Table S7: Demographic and clinic data of EBV+ population; Table S8: List of LMP-1 reference sequences from Genbank; Table S9: List of LMP1 reference and geographic sequences from Genbank to worldwide phylogeny; Figure S1: The overall LMP-1 amino acid (aa) sequence alignment from the sequences generated in this present study (*n* = 82); Figure S2: LMP-1 polymorphisms in the main clades defined in this study.
