# Supplementary material for: Spatial Dispersal of Epstein–Barr Virus in South America Reveals an African American Variant in Brazilian Lymphomas
Source: Viruses. 2022 Aug 12;14(8):1762. doi: 10.3390/v14081762 (PMC9412316; doi:10.3390/v14081762)
Supplement: Supplementary file 1 [file viruses-14-01762-s001.zip › Supplementary Tables S1-S6 Figures S1,S2.pdf]

Table S1 – Phylogenetic classification of population investigated

LMP-1 clade	N (%)	Support (aLRT)
Mediterranean	33 (40.2)	0.84
Raji/Argentine	32 (39.0)	0.99
B95-8	5 (6.1)	0.94
China2	1 (1.2)	0.98
Not classified	11 (13.4)	NA

LMP-1: latent membrane protein 1; N: number of sequences; aLRT: Average Likelihood Ratio test; NA: Not available.

Table S2 – Relationship of LMP-1 clades and clinical outcomes

LMP-1 clade	Clinical outcomes		*p* - value
	ABC N=49	MC N=33	
Mediterranean	23 (46.9)	10 (30.3)	
Raji/Argentine	16 (32.7)	16 (48.5)	
B95-8	2 (4.1)	3 (9.1)	0.3
China2	1 (2.0)	0 (0.0)	
Not classified	7 (14.3)	4 (12.1)	

LMP-1: latent membrane protein 1; N: number of sequences; ABC: Asymptomatic and benign cases; MC: malignant cases.

Table S3 – Chacterization of EBV polymorphisms in the population investigated

Isolate	LMP-1								EBNA3C	Zp
	N-terminus	Transmembrane			C-terminus					
	*Xho*I ctcgag	position 169055 (C>T)	I124V/I152L	F144I/D150A/L151I	n° rep33	ins15 (which rep33)	del30	variant (clade)		
ACBR01	wt	wt	I124V/I152L	wt/wt/L151I	6	yes (4°)	del30	Raji/Argentine	Type 1	NA
ACBR02	wt	wt	wt/wt	wt/wt/L151I	4	no	wt	Raji/Argentine	Type 1	V1
ACBR03	wt	wt	I124V/I152L	wt/wt/L151I	5	yes (4°)	del30	Raji/Argentine	Type 2	V3
ACBR04	wt	wt	wt/wt	wt/wt/L151I	3	no	wt	Mediterranean	Type 1	V1
ACBR05	wt	C>T	wt/wt	F144I/D150A/L151I	5	no	del30	No classified	Type 2	V3
ACBR06	wt	wt	wt/wt	wt/wt/wt	3	no	wt	Mediterranean	Type 1	NA
ACBR07	wt	wt	wt/wt	wt/wt/wt	4	yes (3°)	wt	B95-8	Type 1	V1
ACBR08	wt	wt	wt/wt	wt/wt/wt	4	no	wt	Mediterranean	Type 1	V1
ACBR09	wt	wt	I124V/I152L	wt/wt/L151I	5	yes (4°)	del30	Raji/Argentine	Type 1	NA
ACBR10	wt	wt	wt/wt	wt/wt/wt	5	no	del69	Mediterranean	Type 1	NA
ACBR11	wt	wt	wt/wt	wt/wt/L151I	4	yes (3°)	wt	Raji/Argentine	Type 1	NA
ACBR12	wt	wt	wt/wt	wt/wt/wt	7	no	del30	No classified	Type 1	V1
ACBR13	wt	C>T	wt/wt	F144I/D150A/L151I	5	no	del30	No classified	Type 2	V3
ACBR14	wt	wt	I124V/I152L	wt/wt/L151I	5	yes (4°)	del30	Raji/Argentine	Type 1	V1
ACBR15	wt	wt	I124V/I152L	wt/wt/L151I	5	yes (4°)	del30	Raji/Argentine	Type 1	V1
ACBR16	wt	wt	I124V/I152L	wt/wt/L151I	4	yes (3°)	wt	Raji/Argentine	Type 1	V1
ACBR17	wt	wt	wt/wt	wt/wt/L151I	4	no	wt	Mediterranean	Type 1	V1
ACBR18	wt	wt	wt/wt	wt/wt/L151I	4	no	wt	Mediterranean	Type 1	NA
ACBR19	wt	wt	I124V/I152L	wt/wt/L151I	5	yes (4°)	del30	Raji/Argentine	Type 1	NA

ACBR20	wt	wt	wt/wt	wt/wt/wt	8	no	del30	Mediterranean	Type 1	NA
ACBR21	wt	wt	wt/wt	wt/wt/wt	4	no	del30	No classified	Type 1	NA
ACBR22	wt	wt	I124V/I152L	wt/wt/L151I	5	yes (4°)	del30	Raji/Argentine	Type 2	V3
ACBR23	wt	wt	wt/wt	wt/wt/wt	6	no	wt	Mediterranean	Type 1	V1
ACBR24	wt	wt	I124V/I152L	wt/wt/L151I	5	yes (4°)	del30	Raji/Argentine	Type 1	V3
ACBR25	wt	wt	wt/wt	wt/wt/wt	5	no	wt	Mediterranean	Type 2	V3
ACBR26	wt	wt	wt/wt	wt/wt/wt	6	no	wt	Mediterranean	Type 1	V1
ACBR27	wt	wt	wt/wt	wt/wt/wt	1	no	del69	Mediterranean	Type 2	V3
ACBR28	wt	wt	I124V/I152L	wt/wt/L151I	5	yes (4°)	del30	Raji/Argentine	Type 1	V1
ACBR29	wt	wt	wt/wt	wt/wt/wt	5	no	del69	Mediterranean	Type 2	V3
ACBR30	CTCTAG	wt	wt/wt	F144I/wt/L151I	5	no	wt	China2	Type 1	V3
ACBR31	wt	wt	I124V/I152L	wt/wt/L151I	6	yes (3°)	del30	Raji/Argentine	Type 2	V3
ACBR32	wt	wt	wt/wt	wt/wt/wt	4	no	del30	No classified	Type 1	V1
ACBR33	wt	wt	wt/wt	wt/wt/wt	5	no	wt	Mediterranean	Type 1	V1
ACBR34	wt	wt	wt/wt	wt/wt/L151I	4	no	wt	Mediterranean	Type 1	V1
ACBR35	wt	C>T	wt/wt	F144I/D150A/L151I	4	no	del30	No classified	Type 2	V3
ACBR36	wt	wt	wt/wt	wt/wt/wt	3	no	wt	Mediterranean	Type 1	V1
ACBR37	wt	wt	wt/wt	wt/wt/L151I	4	no	wt	Mediterranean	Type 1	V1
ACBR38	wt	wt	I124V/I152L	wt/wt/L151I	6	yes (4°)	del30	Raji/Argentine	Type 1	V1
ACBR39	wt	wt	wt/wt	wt/wt/L151I	4	no	wt	Mediterranean	Type 1	V1
ACBR40	wt	wt	wt/wt	wt/wt/L151I	6	no	wt	Mediterranean	Type 1	V1
ACBR41	wt	wt	I124V/I152L	wt/wt/L151I	5	yes (3°)	del30	Raji/Argentine	Type 1	V1
ACBR42	wt	wt	wt/wt	wt/wt/L151I	5	no	wt	Mediterranean	Type 1	V1
ACBR43	wt	wt	wt/wt	wt/wt/L151I	5	no	wt	Mediterranean	Type 1	V1
ACBR44	wt	wt	wt/wt	wt/wt/wt	5	yes (2°)	wt	B95-8	Type 2	V3
ACBR45	wt	wt	I124V/I152L	wt/wt/L151I	5	yes (2°)	wt	Raji/Argentine	Type 1	V3
RLHBR01	wt	wt	wt/wt	wt/wt/wt	5	no	del30	No classified	Type 1	NA

RLHBR02	wt	wt	wt/wt	wt/wt/L151I	4	no	wt	Mediterranean	Type 1	NA
RLHBR03	wt	wt	wt/wt	wt/wt/L151I	4	no	wt	Mediterranean	Type 1	NA
RLHBR04	wt	wt	wt/wt	wt/wt/wt	4	no	wt	Mediterranean	Type 1	V1
BLBR01	wt	wt	wt/wt	wt/wt/wt	3	no	wt	Mediterranean	Type 1	V1
BLBR02	wt	wt	wt/wt	wt/wt/L151I	5	no	wt	Mediterranean	Type 1	V1
BLBR03	wt	wt	wt/wt	wt/wt/wt	4	no	wt	Mediterranean	Type 1	V1
BLBR04	wt	wt	I124V/I152L	wt/wt/L151I	4	yes (3°)	del30	Raji/Argentine	Type 1	V1
BLBR05	wt	wt	I124V/I152L	wt/wt/L151I	5	yes (4°)	del30	Raji/Argentine	Type 1	V1
BLBR06	wt	wt	I124V/I152L	wt/wt/L151I	4	no	wt	Raji/Argentine	Type 1	V1
BLBR07	wt	wt	wt/wt	wt/wt/L151I	6	yes (5°)	del30	No classified	Type 1	NA
CHLBR01	wt	wt	wt/wt	wt/wt/wt	4	no	del30	Mediterranean	Type 1	V1
CHLBR02	wt	wt	wt/wt	wt/wt/wt	5	yes (4°)	wt	B95-8	Type 1	V1
CHLBR03	wt	wt	I124V/I152L	wt/wt/L151I	5	yes (4°)	del30	Raji/Argentine	Type 1	V1
CHLBR04	wt	wt	I124V/I152L	wt/wt/L151I	5	yes (4°)	del30	Raji/Argentine	Type 1	V1
CHLBR05	wt	wt	wt/wt	wt/wt/L151I	4	no	wt	Mediterranean	Type 1	V1
CHLBR06	wt	wt	wt/wt	wt/wt/wt	4	yes (3°)	wt	B95-8	Type 2	V3
CHLBR07	wt	wt	wt/wt	wt/wt/L151I	6	yes (5°)	wt	Mediterranean	Type 1	NA
CHLBR08	wt	wt	I124V/I152L	wt/wt/L151I	5	yes (4°)	del30	Raji/Argentine	Type 1	V3
CHLBR09	wt	wt	wt/wt	wt/wt/wt	4	no	wt	Mediterranean	Type 1	V1
CHLBR10	wt	wt	I124V/I152L	wt/wt/L151I	4	yes (3°)	wt	Raji/Argentine	Type 1	V3
CHLBR11	wt	wt	wt/I152L	wt/wt/L151I	4	yes (3°)	wt	No classified	Type 1	NA
CHLBR12	wt	wt	I124V/I152L	wt/wt/L151I	5	yes (4°)	del30	Raji/Argentine	Type 1	NA
CHLBR13	wt	wt	wt/wt	wt/wt/wt	4	yes (3°)	wt	B95-8	Type 1	V1
CHLBR14	NA	wt	I124V/I152L	wt/wt/L151I	5	yes (4°)	del30	Raji/Argentine	Type 1	V1
CHLBR15	wt	wt	I124V/I152L	wt/wt/L151I	4	no	wt	Raji/Argentine	Type 1	V1
CHLBR16	wt	C>T	wt/wt	F144I/D150A/L151I	5	no	del30	No classified	Type 2	NA
CHLBR17	wt	C>T	wt/wt	F144I/D150A/L151I	2	no	del30	No classified	Type 1	V1

CHLBR18	wt	wt	I124V/I152L	wt/wt/L151I	6	yes (4°)	del30	Raji/Argentine	Type 2	V3
CHLBR19	wt	wt	I124V/I152L	wt/wt/L151I	4	yes (3°)	wt	Raji/Argentine	Type 1	V3
CHLBR20	wt	wt	I124V/I152L	wt/wt/L151I	5	yes (4°)	del30	Raji/Argentine	Type 1	V1
CHLBR21	wt	wt	I124V/I152L	wt/wt/L151I	5	yes (4°)	del30	Raji/Argentine	Type 1	V1
CHLBR22	wt	wt	I124V/I152L	wt/wt/L151I	4	no	wt	Raji/Argentine	Type 1	V1
CHLBR23	wt	wt	wt/wt	wt/wt/L151I	4	yes (3°)	wt	Mediterranean	Type 1	V1
CHLBR24	wt	wt	wt/wt	wt/wt/L151I	4	yes (3°)	wt	Mediterranean	Type 1	V1
CHLBR25	wt	wt	wt/wt	wt/wt/wt	4	yes (3°)	wt	Mediterranean	Type 1	NA
CHLBR26	wt	wt	I124V/I152L	wt/wt/L151I	5	yes (4°)	del30	Raji/Argentine	Type 1	NA

LMP-1: latent membrane protein 1; *XhoI*: endonuclease type 2, from *Xanthomonas holcicola*; wt: wildtype; C: cytosine; T: thymine; I: isoleucine; V: valine; L: leucine; F: phenylalanine; D: aspartic acid; A: alanine; rep: repetition; bp: base pairs; ins15: 15 bp insert; del30: 30 bp deletion; del69: 69 bp deletion; NA: Not available.

Table S4 – Relationship of polymorphisms with clades and clinical outcomes investigated

Polymorphisms	LMP-1 clade N (%)					*p - value*		Clinical outcomes N (%)		*p - value*
	Med	Raji	B95-8	Asian	No classified	all	Med x Raji	ABC	MC	
**LMP-1 N-terminus polymorphisms**										
*Xho*I restriction site (c↓tcgag)	33 (100)	31 (100)	5 (100)	0 (0.0)	11 (100)	*p*<0.0001	*p**	48 (98.0)	32 (100)	*p*=1
*Xho*I restriction site lost	0 (0.0)	0 (0.0)	0 (0.0)	1 (100)	0 (0.0)			1 (2.0)	0 (0.0)	
**LMP-1 Transmembrane polymorphisms**										
Position 169055 wt	33 (100)	32 (100)	5 (100)	1 (100)	6 (54.5)	*p*<0.0001	*p**	46 (93.9)	31 (93.9)	*p*=1
Position 169055 C>T	0 (0.0)	0 (0.0)	0 (0.0)	0 (0.0)	5 (45.5)			3 (6.1)	2 (6.1)	
wt/wt	33 (100)	2 (6.2)	5 (100)	1 (100)	10 (90.9)			35 (71.4)	16 (48.5)	
wt/I152L	0 (0.0)	0 (0.0)	0 (0.0)	0 (0.0)	1 (16.7)	*p*<0.0001	*p*<0.0001	0 (0.0)	1 (3.0)	*p*=0.071
I124V/I152L	0 (0.0)	30 (93.8)	0 (0.0)	0 (0.0)	0 (0.0)			14 (28.6)	16 (48.5)	
wt/wt/wt	17 (51.5)	0 (0.0)	5 (100)	0 (0.0)	4 (36.4)			18 (36.7)	8 (24.2)	
wt/wt/L151I	16 (48.5)	32 (100)	0 (0.0)	0 (0.0)	2 (18.2)	*p*<0.0001	*p*<0.0001	27 (55.1)	23 (69.7)	*p*=0.5
F144I/wt/L151I	0 (0.0)	0 (0.0)	0 (0.0)	1 (100)	0 (0.0)			1 (2.0)	0 (0.0)	
F144I/D150A/L151I	0 (0.0)	0 (0.0)	0 (0.0)	0 (0.0)	5 (45.5)			3 (6.1)	2 (6.1)	

**LMP-1 C-terminus polymorphisms**										
33 bp repetition										
x1	1 (3.0)	0 (0.0)	0 (0.0)	0 (0.0)	0 (0.0)			1 (3.0)	0 (0.0)	
x2	0 (0.0)	0 (0.0)	0 (0.0)	0 (0.0)	0 (0.0)			0 (0.0)	1 (3.0)	
x3	4 (12.1)	0 (0.0)	0 (0.0)	0 (0.0)	0 (0.0)			4 (12.1)	1 (3.0)	
x4	16 (48.5)	9 (28.1)	3 (60.0)	0 (0.0)	4 (36.4)	*p*=0.2	*p*=0.01	16 (48.5)	16 (48.5)	*p*=0.5
x5	7 (21.2)	19 (59.4)	2 (40.0)	1 (100)	4 (36.4)			7 (21.2)	12 (36.4)	
x6	4 (12.1)	4 (12.5)	0 (0.0)	0 (0.0)	1 (9.1)			4 (12.1)	3 (9.1)	
x7	0 (0.0)	0 (0.0)	0 (0.0)	0 (0.0)	1 (9.1)			0 (0.0)	0 (0.0)	
x8	1 (3.0)	0 (0.0)	0 (0.0)	0 (0.0)	0 (0.0)			1 (3.0)	0 (0.0)	
Without ins15 bp	29 (87.9)	4 (12.5)	0 (0.0)	1 (100)	9 (81.8)	*p*<0.0001	*p*<0.0001	32 (65.3)	11 (33.3)	*p*=0.007
Ins15 bp	4 (12.1)	28 (87.5)	5 (100)	0 (0.0)	2 (18.2)			17 (34.7)	22 (66.7)	
Ins15 position										
2° 33 bp repetition	0 (0.0)	1 (3.6)	1 (20.0)	0 (0.0)	0 (0.0)			2 (11.8)	0 (0.0)	
3° 33 bp repetition	3 (75.0)	7 (25.0)	3 (60.0)	0 (0.0)	1 (50.0)	*p*=0.003	*p*=0.005	5 (29.4)	9 (40.9)	*p*=0.2
4° 33 bp repetition	0 (0.0)	20 (71.4)	1 (20.0)	0 (0.0)	0 (0.0)			10 (58.8)	11 (50.0)	
5° 33 bp repetition	1 (25.0)	0 (0.0)	0 (0.0)	0 (0.0)	1 (50.0)			0 (0.0)	2 (9.1)	
wt	28 (84.8)	9 (28.1)	5 (100)	1 (100)	1 (9.1)	*p*<0.0001	*p*<0.0001	26 (53.1)	18 (54.5)	*p*=0.3

del30	2 (6.1)	23 (71.9)	0 (0.0)	0 (0.0)	10 (90.9)			20 (40.8)	15 (45.5)	
del69	3 (9.1)	0 (0.0)	0 (0.0)	0 (0.0)	0 (0.0)			3 (6.1)	0 (0.0)	
**Haplotype Type 1 + V3**										
yes	0 (0.0)	5 (19.2)	0 (0.0)	1 (100)	0 (0.0)	*p*=0.003	*p*=0.051	3 (8.1)	3 (11.5)	*p*=0.6
no	25 (100)	21 (80.8)	5 (100)	0 (0.0)	6 (100)			34 (91.9)	23 (88.5)	

LMP-1: latent membrane protein 1; N: number; Med clade: Mediterranean; Raji clade: Raji/Argentine; Asian clade: China1 and China2; ABC: Asymptomatic carrier and benign cases; MC: malignant cases; *Xho*I: endonuclease type 2, from *Xanthomonas holcicola*; wt: wildtype; C: cytosine; T: thymine; I: isoleucine; V: valine; L: leucine; F: phenylalanine; D: aspartic acid; A: alanine; rep: repetition; bp: base pairs; ins15: 15 bp insert; del30: 30 bp deletion; del69: 69 bp deletion; *p**: the variable is constant

Table S5 – Global LMP-1 phylogenetic classification

LMP-1 major clades	Support (aLRT)	Global totality N (%)	LMP-1 minor clades	Support (aLRT)	N (%)						
					Global totality	North America	South America	Europe	Africa	Asia	Oceania
Major clade I	0.96	316 (67)	Mediterranean	0.94	111 (24)	2 (5)	44 (28)	24 (44)	28 (58)	9 (6)	4 (20)
			Raji/Argentine	0.99	91 (19)	5 (12)	72 (45)	5 (9)	8 (17)	0	1 (5)
			B95-8	0.93	43 (9)	13 (32)	11 (7)	9 (16)	2 (4)	5 (4)	3 (15)
			America/Europe	0.75	28 (6)	10 (24)	3 (2)	12 (22)	0	0	3 (15)
			Major clade I not classified	NA	37 (8)	3 (8)	13 (8)	3 (5)	9 (19)	9 (6)	0
Major clade II	0.96	156 (33)	Asian I	0.83	101 (22)	5 (12)	1 (1)	0	0	95 (67)	0
			Asian II	0.95	45 (10)	2 (5)	12 (7)	1 (2)	1 (2)	20 (14)	9 (45)
			Major clade II not classified	NA	8 (2)	1 (2)	2 (2)	1 (2)	0	4 (3)	0

LMP-1: latent membrane protein 1; N: number of sequences; aLRT: Average Likelihood Ratio test; NA: Not available;

Table S6 – Best fit molecular clock and discrete phylogeographic model for the EBV LMP-1 Raji/Argentine clade dataset

Path Sampling	UCLD	Strict	**Log BF (Strict vs UCLD)**
Asymmetric	-3527,4	**-3508,7**	18,6
Symmmetric	-3534,3	-3511,3	23,0
**Log BF (Asym vs Sym)**	6,9	2,6	
Stepping-Stone	UCLD	Strict	**Log BF (Strict vs UCLD)**
Asymmetric	-3528,9	**-3509,0**	19,9
Symmetric	-3535,7	-3511,5	24,2
**Log BF (Asym vs Sym)**	6,8	2,5	

Log marginal likelihood (ML) estimates for strict clock *vs* uncorrelated relaxed clock with a lognormal distribution (UCLD) and Symmetric *vs* Asymmetric discrete phylogeographic models obtained using the path sampling (PS) and stepping-stone sampling (SS) methods. The Log Bayes factor (BF) is the difference of the Log ML between of alternative (H1) and null (H0) models (H1/H0). Log positive BFs values indicates that model H1 is more strongly supported by the data than model H0, while negative values indicate that BF is in favor of the model H0. Each model was systematically compared, and best PS and SS values were highlighted in bold while the best fit model was underlined.

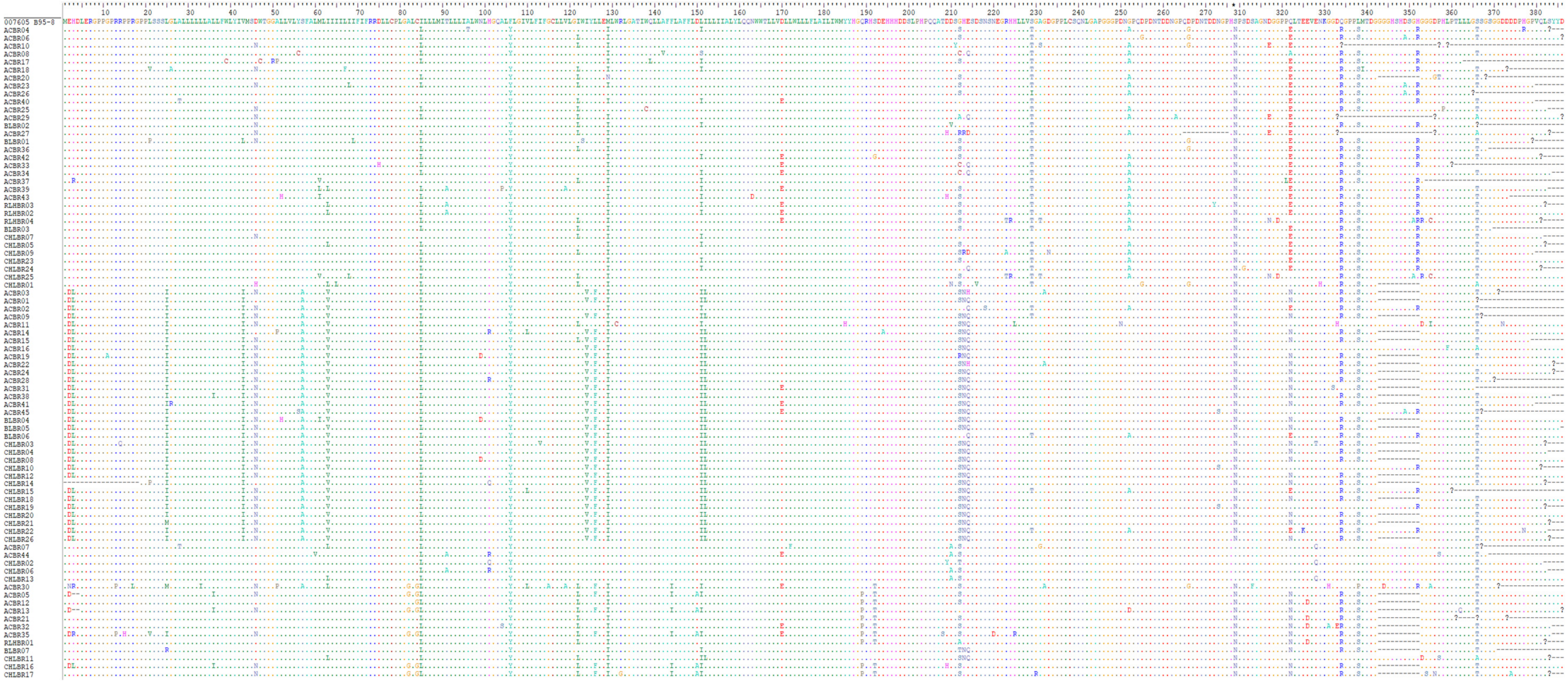

Figure S1 – The overall LMP-1 amino acid (aa) sequence alignment from the sequences generated in this present study (n=82). The alignment order starts with the reference prototype (Genbank number NC_007605) and follows the order of sequences belonging to the Mediterranean clades, followed by those belonging to the Raji/Argentine, B95-8 and Asian II clades, finally the last aligned sequences are those that have not clearly grouped into a specific clade (unclassified). The asterisk represents the cut-off area of the repetitive regions which were not used for the genetic classification of the clades established in this work (nucleotide position -168507 to -168411 from EBV reference prototype genome or 276 to 307 aa in this figure).

(A)

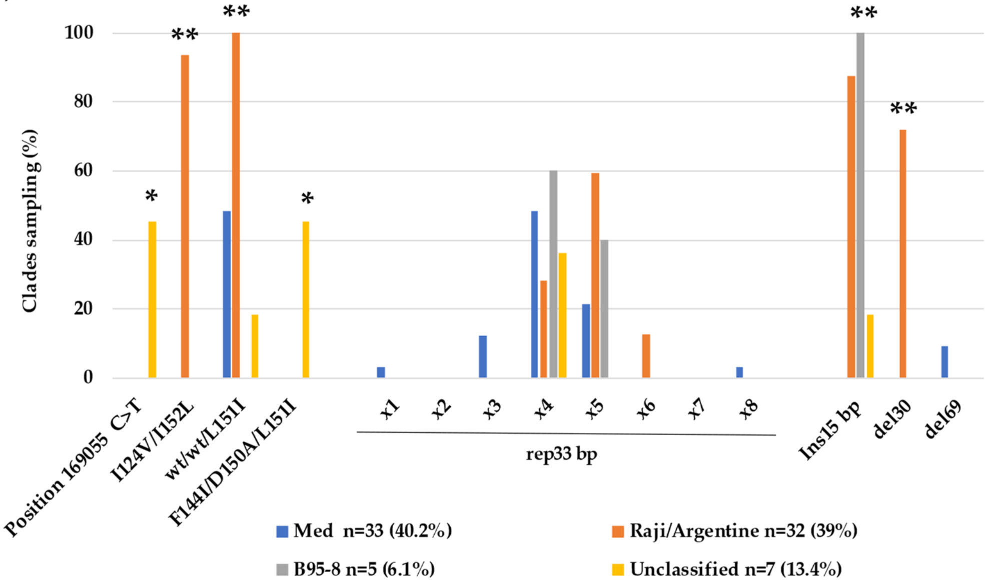

(B)

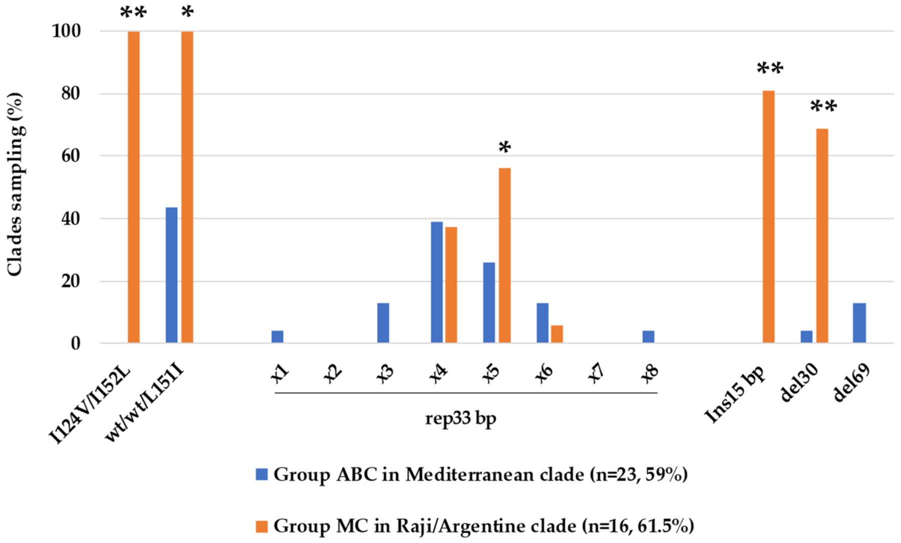

Figure S2 – LMP-1 polymorphisms in the main clades defined in this study. (A) Polymorphisms details in the three main clades defined in this study. The percentage in the subtitle is related to all sequences in this work (n=82), and on the y axis it indicates the sample percentage of each clades. The symbol * indicate $p<0.05$ related to all clades and ** $p<0.05$ when evaluated Mediterranean clade *versus* Raji/Argentine. (B) The relationship of polymorphisms and distinct clinical outcome in the two main LMP-1 lineages circulating in Brazil, thus, the percentage is related to each clinical group that clustered in both clades (ABC, n=39 and MC, n=26). The symbol * indicate $p<0.05$ and ** $p<0.001$. Wt: wildtype; I: isoleucine; V: valine; L: leucine; bp: base pairs; rep33 bp: repeat 33 bp; Ins15 bp: 15 bp insert; del30: 30 bp deletion; del69: 69 base pair deletion.
